# Distinctive Responses in an In Vitro Human Dendritic Cell-Based System upon Stimulation with Different Influenza Vaccine Formulations

**DOI:** 10.3390/vaccines5030021

**Published:** 2017-08-09

**Authors:** Gabriela Tapia-Calle, Maaike Stoel, Jacqueline de Vries-Idema, Anke Huckriede

**Affiliations:** Department of Medical Microbiology, University of Groningen, University Medical Center Groningen, 9713AV Groningen, The Netherlands; m.g.tapia.calle@umcg.nl (G.T.-C.); maaikestoel@gmail.com (M.S.); j.j.de.vries-idema@umcg.nl (J.d.V.-I.)

**Keywords:** dendritic cell, MUTZ-3, whole inactivated virus influenza vaccine, subunit influenza vaccine, flow cytometry, qPCR, cytokines

## Abstract

Vaccine development relies on testing vaccine candidates in animal models. However, results from animals cannot always be translated to humans. Alternative ways to screen vaccine candidates before clinical trials are therefore desirable. Dendritic cells (DCs) are the main orchestrators of the immune system and the link between innate and adaptive responses. Their activation by vaccines is an essential step in vaccine-induced immune responses. We have systematically evaluated the suitability of two different human DC-based systems, namely the DC-cell line MUTZ-3 and primary monocyte-derived DCs (Mo-DCs) to screen immunopotentiating properties of vaccine candidates. Two different influenza vaccine formulations, whole inactivated virus (WIV) and subunit (SU), were used as model antigens as they represent a high immunogenic and low immunogenic vaccine, respectively. MUTZ-3 cells were restricted in their ability to respond to different stimuli. In contrast, Mo-DCs readily responded to WIV and SU in a vaccine-specific way. WIV stimulation elicited a more vigorous induction of activation markers, immune response-related genes and secretion of cytokines involved in antiviral responses than the SU vaccine. Furthermore, Mo-DCs differentiated from freshly isolated and freeze/thawed peripheral blood mononuclear cells (PBMCs) showed a similar capacity to respond to different vaccines. Taken together, we identified human PBMC-derived Mo-DCs as a suitable platform to evaluate vaccine-induced immune responses. Importantly, we show that fresh and frozen PBMCs can be used indistinctly, which strongly facilitates the routine use of this system. In vitro vaccine pre-screening using human Mo-DCs is thus a promising approach for evaluating the immunopotentiating capacities of new vaccine formulations that have not yet been tested in humans.

## 1. Introduction 

Vaccines are the most cost-effective way to battle infectious diseases. Their routine use has enabled the eradication of infectious diseases like smallpox and rinderpest, and the reduction of others like measles and pertussis [1]. However, the development of new vaccines is both expensive and time-consuming, with rarely successful outcomes. Currently, the use of animal models is a key step in assessing vaccine safety and efficacy. However, the use of animals in preclinical studies has disadvantages. Apart from the intrinsic ethical issues that come with their use, animal models cannot completely mimic the immune system of humans [2]. Frequently, vaccine candidates that succeeded in animal experiments fail to do so in clinical trials, this being one of the greatest bottlenecks when screening vaccines [3,4]. Given the limitations of the current animal models, the use of alternative tools to predict vaccine behavior in humans could increase the odds of success of vaccine candidates in clinical trials.

Cultures of antigen-presenting cells (APCs) could provide insights into how the human immune system responds to a vaccine batch or a new vaccine candidate. In the past, human primary cells differentiated to APCs or cell lines representing APCs have allowed us to model and to gain insight into basic immunological responses, e.g., the differentiation of monocytes into APCs, the induction of antigen-specific T helper and memory cells, and the induction of antigen-specific antibody responses (reviewed in [5,6]). An important type of APCs is the dendritic cell (DC), which acts as sentinel of the immune system by detecting the presence of pathogens or vaccines. The key functional features of DCs are to capture, process and present antigens and to deliver accessory signals thereby enabling T cell priming [7]. DCs have a pivotal role in the induction of immune responses, their effects on other immune cells (both innate and adaptive) have great consequences for the adaptive immune response after vaccination [8].

There are two possible sources of human DCs for the investigation of vaccines in vitro: DC-like cell lines and primary DCs isolated and differentiated from peripheral blood mononuclear cells (PBMCs). Generally, the use of cell lines to evaluate immune responses is very appealing since cell lines are a consistent source of stable cells, they are readily available, and they are not subject to variation. However, cell lines have accumulated mutations, amongst others mutations enabling their continuous growth, and thus may no longer respond to triggers in the same way as normal cells would [9]. Primary DCs, on the other hand, require a laborious process of isolation of monocytes from PBMCs and their subsequent differentiation to DC; the sources for PBMCs are limited and more importantly, responses of PBMC-derived DCs might be prone to donor-to-donor variation [10,11]. Notwithstanding, primary cells retain many of the characteristics of the cells in vivo which may translate into a more accurate response than the that obtained with cell lines. 

As a first step towards the development of an in vitro system suitable for pre-screening of vaccine candidates, we set out to identify the best cellular platform to assess in vitro stimulatory properties of vaccine formulations. We have evaluated the responses upon stimulation with vaccines in a DC-like cell line, and in human monocyte-derived DCs (Mo-DCs). As a representative of DC-like cell lines we chose MUTZ-3, a human acute myeloid leukemia cell line. The differentiation of this myeloid cell line to a DC phenotype is dependent on cytokines [12,13] rather than on lipopolysaccharide (LPS) stimulation or pharmacological agents as required for other cell lines. Thus, the conditions MUTZ-3 cells need for differentiation resemble the conditions used to obtain monocyte-derived DCs from primary cells, which confers MUTZ-3 an advantage over other APC-like cell lines [14]. With respect to Mo-DCs, we determined whether freshly isolated and frozen/thawed PBMCs are equally suitable for generation of Mo-DCs and whether Mo-DCs derived from fresh and frozen PBMCs respond to vaccines and do so in a similar way. The use of frozen/thawed PBMCs has so far not been systematically evaluated. Frozen PBMCs as source of monocytes for DC differentiation would make the use of a human primary DC-based platform much more practical, reproducible and efficient. In addition, we evaluated the extent of donor-to-donor variation in the response to vaccines which has so far gained limited attention.

As model antigens we used two different non-adjuvanted influenza vaccine formulations with well-characterized differences in immunogenicity: whole inactivated virus (WIV) and subunit (SU) vaccine. WIV vaccines, produced by inactivation of live virus with formaldehyde or β-propiolactone [15], retain the native virus structure, contain all viral proteins, and possess fusion activity. They also contain single-stranded RNA (ssRNA), which triggers toll-like receptor (TLR) 7 and thus provides a “self-adjuvanted” feature to WIV [16,17]. These characteristics make WIV more immunogenic than SU. The SU vaccine is a highly purified formulation consisting of the viral surface antigens hemagglutinin (HA) and neuraminidase (NA). This vaccine is produced by purification of HA and NA from an inactivated detergent-disrupted influenza virus [18]. Intrinsic differences in the immune responses elicited by the two vaccine formulations have been reported previously in animal models as well as in unprimed humans [19]. Compared to SU vaccines, WIV vaccines have shown to induce higher immonuglobulin G2a (IgG2a) levels against HA and NA [16], higher hemagglutination inhibition titers, a Th1-type (rather than a Th2-type) response [20] and cross-protective cytotoxic T cells in immunized mice [21]. The in vivo results were reflected by in vitro results, demonstrating that WIV induces in mouse DCs a Th1 cytokine profile and prominent changes in the expression of genes involved in antiviral pathways, whereas SU did not [22]. 

Here we report that human Mo-DCs respond to WIV and SU vaccines in a vaccine-specific manner whilst the MUTZ-3 cell line does not. We demonstrate that DCs differentiated from freshly isolated or cryopreserved PBMCs show similar capacities to respond to different vaccines. This enables the indistinct use of fresh and frozen PBMCs for DC generation, thus facilitating the routine use of this DC-based in vitro system. DCs derived from different donors responded to the vaccines in a qualitatively similar way and differed only marginally in quantitative terms for most of our readouts. The ability of cultured DCs to recapitulate vaccine-specific activation patterns upon stimulation with different vaccines represents a pivotal requirement for the development of an in vitro screening platform for the evaluation of novel vaccine formulations. 

## 2. Materials and Methods 

### 2.1. Vaccines 

WIV and SU were produced from the H5N1 virus subtype (NIBRG-14, a 2:6 recombinant of A/Vietnam/1194/2004 (H5N1) and A/PR/8/34 (H1N1) virus produced by reverse genetics technology, obtained from the NIBSC) as described before [16]. Briefly, H5N1 was propagated on embryonated chicken eggs and inactivated with 0.1% β-propiolactone for 24 h at 19–21 °C, followed by dialysis and filtration (0.45 µm) to obtain WIV. SU was produced by solubilizing the β-propiolactone-inactivated virus with the detergent octa(ethyleneglycol)-n-dodecyl monoether (C_12_E_8_) (Nikkol, Tokyo, Japan). Nucleocapsids were removed by ultracentrifugation and membranes were reconstituted by extraction of C_12_E_8_ using Biobeads (Bio-Rad, Hercules, CA, USA). Formed virosomes were concentrated by ultra-centrifugation on a 50% sucrose cushion in Hepes and then processed into subunit vaccine.

### 2.2. Generation of DC Derived from the MUTZ-3 Cell Line 

The human leukemic cell line MUTZ-3 was obtained from the Leibniz-Institute DSMZ (Deutsche Sammlung von Mikroorganismen und Zellkulturen, Braunschweig, Germany) and cultured at 37 °C with 5% CO_2_ in Minimal Essential Medium-α (MEM-α) (Gibco, Life Technologies, Paisley, UK) supplemented with 20% heat-inactivated fetal calf serum (FCS) (Lonza, Basel, Switzerland), 1% penicillin/streptomycin and 500 U/mL granulocyte-macrophage colony-stimulating factor (GM-CSF) (ProsPec; Rehovot, Israel). Cells from passage numbers 10 to 25 were used for differentiation to a DC phenotype. Differentiation conditions tested are summarized in Appendix (Table A1). The culture medium was replaced every 3 days. Cells were seeded at a density of 2 × 10^5^ cells/mL in 12-well plates (Corning, New York, NY, USA) for flow cytometry analysis.

### 2.3. Generation of Immature Mo-DCs from Primary Cells

PBMCs were isolated from buffy coats (Sanquin, Groningen, The Netherlands). Aliquots containing 12.5 mL of buffy coat were mixed with 37.5 mL of RPMI-1641 (Gibco, Life Technologies; Paisley, UK). The mixture was layered on 16 mL of Ficoll Paque (GE Healthcare, Upssala, Sweden) and the tubes were centrifuged at 400× *g* for 30 min at 21 °C without brake. PBMC fractions were collected, pooled, resuspended in RPMI and centrifuged at 250× *g* for 10 min. Red blood cells were lysed with ACK lysis buffer (156 mM NH_4_Cl, 10 mM KHCO_3_, 0.1 mM Na_2_EDTA; pH 7.3) and washed with RPMI. Cells were centrifuged at 250× *g* for 10 min, resuspended in 5 mL RPMI + 5% FCS and viability was determined by Trypan Blue (Gibco, Rockville, MD, USA). At this stage, cells were either used directly for isolation of monocytes (see below) or were frozen. 

PBMCs were placed in a cell freezing container (CoolCell LX, Biocision, Menlo Park, CA, USA) and stored in liquid nitrogen at a concentration of 40 × 10^6^ PBMCs/mL per cryotube in FCS (90%) + dimethyl sulfoxide (DMSO) 10%. Cryotubes containing frozen PBMCs were placed in a water bath at 37 °C until cells were thawed. Cells were pipetted into 15-mL tubes and a 2-fold volume of warm FCS (37 °C) was added slowly. After centrifugation at 500× *g* for 10 min, cells were washed 2 times with washing buffer (phosphate-buffered saline (PBS), 2% FCS, 1 mM ethylenediaminetetraacetic acid (EDTA)), centrifuged and resuspended in washing buffer. Viability was checked with Trypan Blue.

Monocytes from fresh or frozen PBMCs were isolated using an immunomagnetic negative selection kit, the EasySep Human Monocyte Enrichment Kit (Stemcell Technologies, Vancouver, BC, Canada). To obtain dendritic cells, monocytes were seeded at a density of 1 × 10^6^ cells/mL and cultured at 37 °C with 5% CO_2_ in RPMI-1640 medium (L-glutamine, HEPES) supplemented with 10% FCS, 1% penicillin/streptomycin, GM-CSF (450 U/mL) and interleukin-4 (IL-4) (500 U/mL) (ProsPec, Rehovot, Israel). Fresh cytokines were added every 2 days for 6 days.

### 2.4. Treatments

#### 2.4.1. Undifferentiated MUTZ

To check on the MUTZ-3 phenotype before differentiation, cells were stimulated with lipopolysaccharide (LPS; 1 μg/mL; Invivogen, Toulouse, France), imidazoquinoline compound (R848; 10 μg/mL; Invivogen, Toulouse, France), tumor necrosis factor alpha (TNF-α; 2 μg/mL; PeproTech, London, UK), whole inactivated influenza virus (WIV; equivalent to 10 μg/mL HA), subunit influenza vaccine (SU; equivalent to 10 μg/mL HA) or PBS (Gibco, Bleiswijk, The Netherlands) for 24 or 48 h.

#### 2.4.2. MUTZ-3-Derived DCs

After differentiation, stimulation was performed for 24 h with LPS (1 μg/mL), R848 (5 μg/mL), WIV (equivalent to 10 μg/mL HA), SU (10 μg/mL) or TNF-α (2 μg/mL; PeproTech, Rocky Hill, NJ, USA). MUTZ-3 cells were seeded at a density of 2 × 10^5^ cells/mL in 12-well plates.

#### 2.4.3. Monocyte-Derived DCs

After differentiation, immature DCs were exposed for 4 or 24 h to WIV or SU vaccines, R848, or PBS as described above. Cells were seeded at a concentration of 5 × 10^5^ cells/mL per treatment in 12-well plates for qPCR, cytokine and flow cytometry analysis

### 2.5. Surface Marker Staining and Flow Cytometry Analysis

To examine the expression of surface markers associated with the DC phenotype, 2 × 10^5^ cells of each condition were harvested after exposure. Cells were fixed in 200 μL of 4% paraformaldehyde (Merck KGaA, Darmstadt, Germany) in PBS and washed with cold washing buffer (1× PBS supplemented with 2% FCS and 1 mM EDTA). Cells were stained for 45 min at 4 °C in the dark, washed, and resuspended in 200 μL washing buffer. The following labeled mouse anti-human antibodies (all from BD Pharmingen, San Diego, CA, USA) were used for flow cytometry analysis: FITC-MHCII, PE-CD86, APC-CD11c, FITC-CD40, PE-CD80, and APC-CD14. Flow cytometry was performed on a FACS Calibur flow cytometer (BD Pharmingen, San Diego, CA, USA). Data were collected using the Cell Quest software (Becton Dickinson) and analyzed using the Kaluza software (Beckman Coulter, Woerden, The Netherlands) and FlowJo (Tree Star, Inc., Ashland, OR, USA). Isotype controls were used for all of antibodies used to control for aspecific binding and were negative in all cases. 

### 2.6. RNA Isolation and qPCR

Cells from each exposure condition (see results section for details) were centrifuged (1000 rpm, 5 min), resuspended in RNAlater (Qiagen, Hilden, Germany) and stored at −80 °C. RNA isolation was performed using the RNeasy Mini Kit (Qiagen, Hilden, Germany) following the instructions of the manufacturer. For Mo-DCs gene expression of *CD86*, *CD40*, *IL-10*, *MYD88*, *IRF7* and *STAT1* was assessed. Gene expression levels were normalized against *GAPDH* and were quantified relative to time-matched mock stimulated cell cultures. Data were analyzed according to the comparative Ct method [23] and are expressed as fold change.

### 2.7. Cytokine Quantification by Multiplex Immunoassay

Cytokine production was determined by Luminex technology. Dendritic cell supernatants were collected at 24 h after stimulation with the different vaccines. Samples were centrifuged and stored at −80 °C until analysis. The Th1–Th2 cytokine panel containing beads for IL-4, Il-5, IL-6, IL-12p70, TNF-α and interferon (IFN)-γ was supplemented with the single-plexes for IL-1β, Il-2, IL-8 and IL-10 (all from ProcartaPlex, eBioscience). Briefly, 50 µL of undiluted cell supernatant were added to the antibody magnetic beads in each of the 96-well plate-wells for 60 min at room temperature. Each sample was added in duplicate. Next, detection antibodies were added to each well and incubated for 30 min, followed by streptavidin phycoerythrin incubation (50 μL per well). After 30 min of incubation the beads-sample mixtures were resuspended in Reading Buffer (25 µL) and run on a Luminex 100 analyzer (Luminex Corporation, Austin, TX, USA) following the manufacturer’s instructions. Calculations were performed using STarStation software (Applied Cytometry Systems, Sheffield, UK). Data were logarithmically transformed before analysis.

### 2.8. Statistics

Significant differences between the two vaccines (WIV and SU) were determined using the unpaired Student’s *t* test. A *p* value of *p* < 0.05 was considered significant. 

## 3. Results

### 3.1. MUTZ-3 Cells Are Restricted in Their Ability to Respond to Different Stimuli

**Figure 1:**
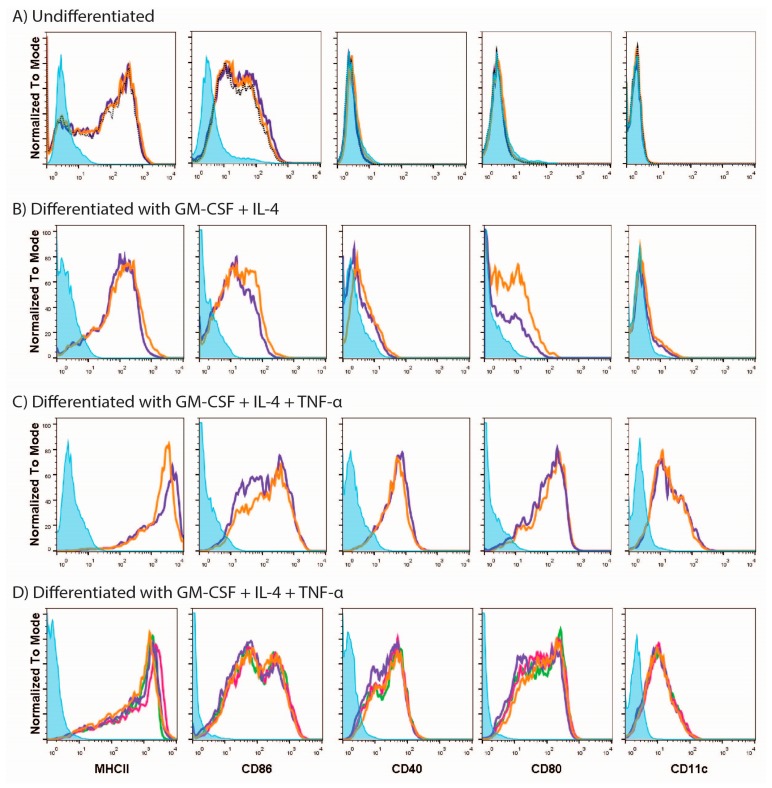
MUTZ-3 needs tumor necrosis factor alpha (TNF-α) for differentiation and does not respond to stimuli. (**A**) Undifferentiated MUTZ-3 cells were assessed for the expression of surface markers prior to differentiation. Histograms show flow cytometry of non-stimulated (dashed black lines), lipopolysaccharide (LPS)-stimulated (orange lines) and mock-stimulated with phosphate-buffered saline PBS (purple lines) cells; (**B**,**C**) Cells were differentiated with granulocyte-macrophage colony-stimulating factor (GM-CSF) + interleukin-4 (IL-4) for 7 days in the absence (**B**) or presence (**C**) of TNF-α and stimulated for 24 h with LPS (orange lines) or mock-stimulated with PBS (purple lines); (**D**) Cells differentiated for 7 days were stimulated for 24 h with LPS (orange), imidazoquinoline compound(R848) (green), TNF-α (pink) or PBS (purple). Blue filled peaks represent isotype controls.

In order to find a suitable platform for assessing vaccines in vitro we initially turned to MUTZ-3 cells, a cell line similar to primary monocytes in its dependence on cytokines for differentiation to DCs. We first analyzed the phenotype of the MUTZ-3 cell line prior to differentiation and assessed whether undifferentiated MUTZ-3 cells could respond to stimuli (Figure 1, panel A). Flow cytometric analysis showed that undifferentiated MUTZ-3 cells expressed MHCII and CD86 but not CD11c, CD80 or CD40 (Figure 1A, purple lines) indicating that these cells do not have a DC phenotype. As expected, undifferentiated MUTZ-3 cells did not respond to LPS, known as a strong trigger of DC activation (Figure 1A, orange lines).

We next tried to differentiate the cells to a dendritic cell phenotype by using various conditions. Surface marker evaluation indicated that differentiation of MUTZ-3 cells with GM-CSF and IL-4 (Figure 1, panel B) resulted in minor up-regulation of CD11c, a DC phenotype marker (compare purple lines in Figure 1A,B). Furthermore, there was up-regulation in the expression of CD40 and CD80 and a more homogenous expression of MHCII but little change in the expression level of CD86. The limited changes in surface marker expression are in line with microscopical observations indicating that MUTZ-3 cells differentiated with GM-CSF and IL-4 alone have the same morphological appearance as undifferentiated cells (data not shown). However, when TNF-α was included in the cytokine mix used for differentiation (Figure 1, panel C) there was up-regulation of CD11c, demonstrating that under those conditions the cells differentiated to a DC phenotype (Figure 1C). Up-regulation of CD11c went along with enhanced expression of MHCII, CD86, CD40 and CD80 as compared to expression on undifferentiated cells. Morphological observations revealed long dendrites, consistent with a DC-like phenotype in differentiated MUTZ-3 cells (data not shown). Thus, successful differentiation was TNF-α-dependent. 

Responsiveness of successfully differentiated MUTZ-3 cells to external stimuli was evaluated by exposing the cells to LPS. Unexpectedly, surface marker expression of cells stimulated with LPS or mock-stimulated with PBS did not differ for any of the markers studied (Figure 1C, orange lines). 

Unresponsiveness to LPS could be due to a defective function of the LPS itself or to the fact that the matured status of the DCs was already reached with no possibility of further activation. We sought to discriminate between these possibilities by using other stimuli. We exposed the cells to a different batch of LPS, to the TLR7 ligand R848, or to an additional dose of TNF-α. However, 24 h after stimulation we did not find any difference between the stimulated and mock-stimulated cells in terms of expression of surface markers for any of the stimuli (Figure 1, panel D). Similarly, neither WIV nor SU vaccines had any effect on the expression profile of the surface markers.

From the above results we concluded that differentiated MUTZ-3 cells are refractory to triggering with toll-like receptor (TLR) ligands or viral vaccines and are thus not suitable for testing responses to vaccines in vitro.

### 3.2. Mo-DCs Do Respond to External Stimuli

**Figure 2:**
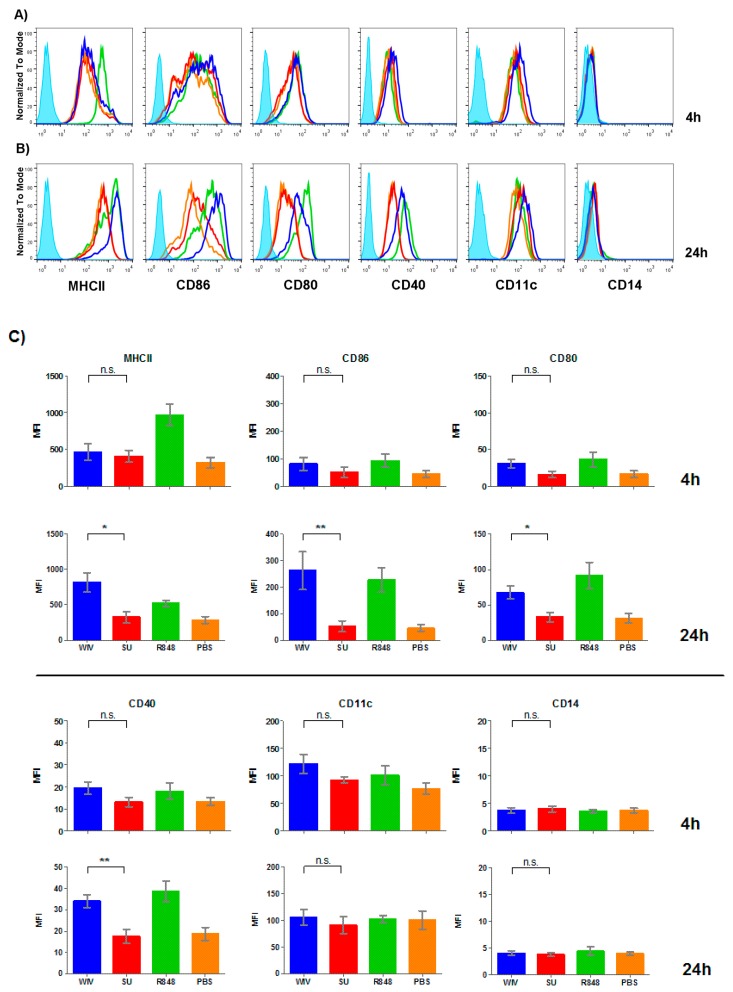
Monocyte-derived dendritic cells (Mo-DCs) show a different surface marker profile when stimulated with different vaccines. Immature DCs were stimulated for 4 h (**A**) and 24 h (**B**) with whole inactivated virus (WIV; blue), subunit (SU; red), R848 (green) or mock-stimulated with PBS (orange). Blue filled peaks represent isotype controls. Subsequently cells were analyzed by flow cytometry for the expression of maturation and activation markers. Histograms correspond to one representative donor (*n* = 4); (**C**) Bar graphs depicting mean fluorescent intensities (MFIs) and standard deviations for the different surface and activation markers; *n* = 4. Levels of significance: not significant (n.s.): *p* > 0.05; *: *p* ≤ 0.05; and **: *p* ≤ 0.01.

Since the MUTZ-3 cell line proved unsuitable for the evaluation of responses to external stimuli and vaccines, we next turned to Mo-DCs. Freshly isolated monocytes from PBMCs of healthy donors were differentiated to DCs by exposure to GM-CSF and IL-4 for a period of 6 days. Successful differentiation was confirmed by lack of CD14 expression and expression of the myeloid marker CD11c (Figure 2). Flow cytometric analysis further revealed that the differentiated DCs had an immature phenotype characterized by expression of low levels of MHCII and no expression of CD86, CD40, nor CD80. Stimulation of Mo-DCs with R848 (positive control) already induced MHCII expression at the 4 h time point while the rest of the markers did not show early changes in expression level (Figure 2, panel A, compare green line (R848) with orange line (PBS control). After 24 h of stimulation with R848, we found induction of MHCII, CD86, CD80 and to a lesser extent CD40 (Figure 2B). Mo-DCs were thus able to efficiently respond to a synthetic stimulus. 

Next we analyzed the response induced by the two different influenza vaccine formulations. When Mo-DCs were stimulated with SU vaccine we did not find any significant up-regulation of any of the markers after 4 h or after 24 h (Figure 2, red line). As a general trend, the SU stimulation profile always mimicked the profile of the mock-stimulated cells. In contrast, WIV already elicited up-regulation of MHCII at the 4 h time point. After 24 h of stimulation with WIV, there was a significant up-regulation of MHCII, CD86, CD80 and CD40, consistent with an activated DC phenotype (Figure 2B, green line). This phenotype was similar to that of cells stimulated with R848. Differential expression of surface markers elicited by WIV and SU was consistently found for Mo-DCs derived from four different donors with only minor variation, reflected in the relatively small standard deviations (Figure 2C). Thus, Mo-DCs responded in characteristic ways to SU and WIV and the responses were in line with the magnitude of the immune responses these vaccine evoke in vivo.

**Figure 3:**
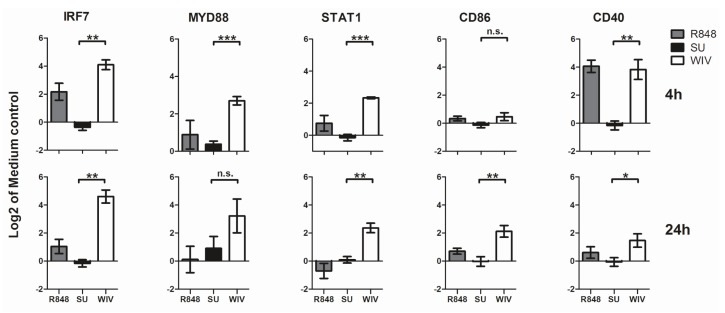
Gene expression shows that Mo-DCs respond to stimulation with R848 and two different influenza formulations in distinctive ways. Immature DCs were stimulated with WIV, SU and R848 for 4 h (upper panel) and 24 h (lower panel), RNA was isolated and gene expression was assessed by reverse transcription polymerase chain reaction (RT-qPCR). Bars represent the mean log2 fold change in gene expression as compared to the medium control of four different donors +/− standard deviation. Levels of significance: n.s.: *p* > 0.05; *: *p* ≤ 0.05; **: *p* ≤ 0.01 and ***: *p* ≤ 0.001.

To further evaluate the ability of Mo-DCs to respond to stimuli and to different vaccines in a characteristic way, we assessed the expression of selected genes involved in the immune response by reverse transcription polymerase chain reaction (RT-qPCR). For this, we chose genes coding for the adaptor protein *MYD88*, a transcription factor involved in the TLR signaling pathway (*IRF7*), a transcription factor involved in the response to interferons (*STAT1*) and genes coding for surface markers relevant for the DC phenotype (*CD40* and *CD80*) (Figure 3). Our results reveal that 4 h stimulation with R848 induced up-regulation in the gene expression levels of *CD40* and to a lower extent *IRF7*, while the other investigated genes showed less than 2-fold changes in expression. After 24 h of stimulation, the expression levels of most genes were back to the levels in control cells. SU vaccine did not successfully stimulate the Mo-DCs. This was reflected by the absence of an evident up- or down-regulation of any of the genes at 4 h and 24 h. WIV-stimulated cells showed more than 2-fold upregulation compared to the mock-stimulated controls in all of the investigated genes. The difference in fold change of gene expression between WIV- and SU-stimulated cells was significant for most of the genes except for *CD86* at the 4 h time point and *MYD88* at the 24 h time point. The fold increase in induction elicited by WIV was higher than the one elicited by the synthetic *TLR7* ligand R848. While the expression of most markers had returned to normal levels after 24 h of R848 stimulation the increase in marker expression was sustained in cells stimulated with WIV. All four investigated donors responded in the same way and to a similar extent to stimulation with R848, SU and WIV as reflected in the relative small standard deviations shown in Figure 3.

**Figure 4:**
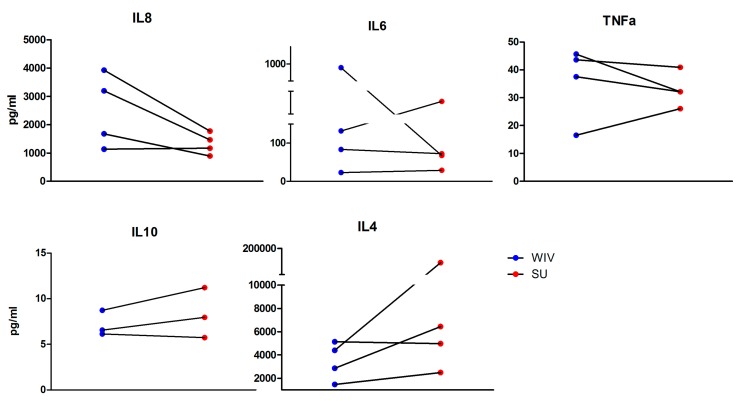
Stimulation of Mo-DCs with WIV elicits a higher production of cytokines than stimulation with SU. Supernatants from WIV, SU and mock-stimulated cells were collected 24 h after stimulation. Cytokines secreted were detected with a multiplex immunoassay using Luminex technology. Cytokine concentrations are given in pg/mL. Blue and red dots correspond to cells stimulated with WIV and SU, respectively. Dots connected with a line were obtained with cells from the same donor.

We additionally tested the effect of R848 and the two vaccine formulations on the production of cytokines by Mo-DCs. Cytokines were chosen based on their ability to polarize the immune response towards a Th1- or Th2-like phenotype or to reflect or induce an antiviral response. We were particularly interested in IL-2, IL-12p70, IFN-γ, IL-1β, IL-10, IL4, and TNF-α, given their involvement in the immune response during influenza infection and also given the phenotype of the response upon vaccination. However, IL-2, IL-12p70, IFN-γ, and IL-1β were not produced by DCs exposed to either of the vaccines. IL-8, a neutrophil and T cell-chemoattractant, was produced more effectively in DCs exposed to WIV than in DCs exposed to SU, at least in the cells of three of the four donors studied (Figure 4). TNF-α and IL-6, both involved in inflammatory responses, showed relatively few differences in their production when DCs were stimulated with WIV or SU. Lastly, IL-10 and IL-4, which are mainly associated with Th2 responses, showed a marginally higher production when DCs were stimulated with SU as compared to WIV, which is in line with the known Th2 immune phenotype evoked by SU. 

In general, we found a high extent of donor-to-donor variation for cytokine expression, reflected in low and high responders for most of the cytokines measured (IL-8, IL-6, TNF-α and IL-4) (Figure 4). Despite this variation, we could still see differences in the cytokine profile depending on the vaccine being used. 

Taken together, Mo-DCs were able to respond to synthetic stimuli and vaccines by changes in the expression of surface and activation markers, expression of immune-related genes and the secretion of cytokines. Above all, the Mo-DCs responded in characteristic ways to different vaccines. Overall, stimulation with WIV resulted in upregulation of surface markers and induction of immune-related genes while stimulation with SU did not or did so to a lower extent.

### 3.3. Mo-DCs Derived from Freshly Isolated and Frozen/Thawed PBMCs Are Similar in Their Capacity to Respond to Stimuli 

PBMC isolation is a long process and access to buffy coats or blood can be sometimes restricted. In order to assess whether cryopreserved PBMCs could substitute for freshly isolated PBMCs we compared the responses of Mo-DCs differentiated from freshly isolated and from frozen/thawed PBMCs of the same donors using the same stimuli and readouts as previously described. Immature DCs were stimulated with R848, SU, or WIV, or were mock-stimulated with PBS. After 4 h and 24 h, stimulation cells were harvested for flow cytometry, RT-qPCR and multiplex immunoassay. 

**Figure 5:**
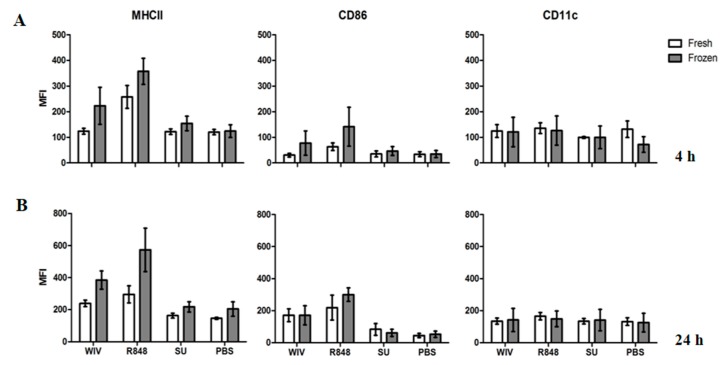
Mo-DCs obtained from freshly isolated and frozen/thawed peripheral blood mononuclear cells (PBMCs) are equally able to upregulate expression of surface markers upon stimulation with WIV or R848. Monocyte-derived DCs from freshly isolated and frozen/thawed PBMCs of matching donors were differentiated for 6 days and stimulated with WIV, R848, SU and PBS for 4 h (**A**) and 24 h (**B**). Bars represent the MFI for MHCII, CD86 and CD11c of four different donors +/− standard deviation.

Firstly, we focused on the expression of surface markers on Mo-DCs derived from fresh or frozen/thawed cells (Figure 5). Importantly, mean fluorescent intensities (MFIs) for the investigated markers did not differ significantly in the mock-stimulated cells derived from fresh or thawed PBMCs. This indicated that the freeze/thawing process as such did not induce activation of the cells. We then compared the MFIs of Mo-DCs stimulated with WIV or R848. Both stimuli were able to activate the cells, which was seen in the upregulation of MHCII and CD86 as compared to the mock-stimulated control at the 24 h time point. The phenotypes and the extents of the responses to either of the stimuli were consistent between DCs derived from fresh and frozen cells. Similarly to PBS, SU stimulation did not induce a significant expression of any surface marker (Figure 5). 

**Figure 6:**
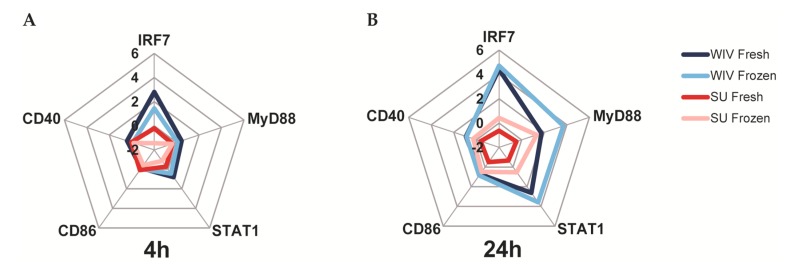

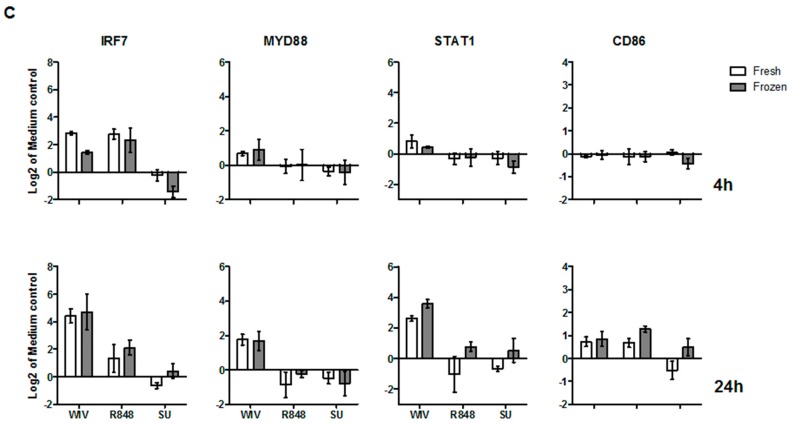
Differences in gene expression induced by WIV and SU are consistent in Mo-DCs derived from fresh and frozen/thawed PBMCs. (**A**,**B**) WIV––(blue lines) and SU––(red-lines) induced changes in gene expression in Mo-DCs derived from fresh (dark colors) and frozen/thawed (light colors) PBMCs from matching donors. Cells were stimulated for 4 h (**A**) and 24 h (**B**). Results are expressed as mean log2 fold change compared to mock-stimulated cells (*n* = 4); (**C**) Quantitative representation of the gene expression data. Bars represent the mean log2 fold change in gene expression as compared to the medium control of four different donors +/− standard deviation.

Gene expression profiling revealed similar expression profiles for Mo-DCs differentiated from freshly isolated and frozen/thawed PBMCs. As can be deduced from the radar plots in Figure 6A,B and the bar graphs in Figure 6C, Mo-DCs showed similarly low responses to SU after 4 h as well as after 24 h of stimulation irrespective of whether they were derived from fresh or frozen PBMCs (dark red and light red lines). The cells also responded in a similar way to WIV and these responses were much stronger than those to SU (dark blue and light blue lines). Irrespective of the vaccine, Mo-DCs derived from frozen/thawed PBMCs exhibited somewhat higher gene induction levels after 24 h of stimulation than those derived from freshly isolated PBMCs. 

Finally, we assessed whether the source of the PBMCs affected the production of cytokines by Mo-DCs. Though we found large individual variation in the amount of cytokines expressed, Mo-DCs derived from fresh or frozen PBMCs of a particular donor showed similar baseline levels for the cytokines measured (data not shown). We also found donor related differences in the up-regulation of cytokine expression upon exposure of the cells to vaccine. Again, these were consistent regardless whether the DCs were derived from fresh or frozen/thawed cells. 

Thus, both fresh and frozen PBMCs are equally suitable for the generation of Mo-DCs and these cells can respond in characteristic ways to synthetic stimuli and vaccines.

## 4. Discussion

In order to develop an in vitro system for pre-screening of vaccine candidates, we have set out to identify a suitable human cellular platform to assess responses towards vaccines using WIV and SU influenza vaccines as model antigens. We demonstrate that the MUTZ-3 cell line does not show any reaction to the vaccines and is thus unsuitable as a platform for an in vitro vaccine screening system. In contrast, human primary Mo-DCs did respond to exposure to the vaccines. More importantly, as previously described for mouse bone marrow-derived cells, Mo-DCs responded in a vaccine-specific manner and the response was in agreement with the known in vivo immunogenicity of the two vaccines [22,24,25]. The ability to respond distinctly towards different vaccine formulations was not hampered when Mo-DCs were derived from frozen PBMCs. Overall, the response obtained across DCs from a number of different donors was consistent. To our knowledge, we are the first to systematically compare responses to stimuli in cells derived from fresh and frozen cells and to carefully assess variation in responses in cells derived from different donors across different read-outs. Our results support the feasibility of using an in vitro approach using human PBMC-derived DCs to estimate in vivo responses elicited by different vaccine formulations.

Recently, the MUTZ-3 cell line has been proposed as a suitable DC in vitro model [14,26,27] and potential tool to investigate features of DC biology and immunology. However, while some groups have claimed this cell line mimics primary DCs in different aspects such as phenotype, transcriptional profile and their capacity to induce T cell proliferation [14,27,28,29,30,31]; others have reported impaired responsiveness to certain stimuli and transcriptional and functional defects in this cell line [5,9,32,33]. Regardless, when compared to other DC-like cell lines (THP-1, Monomac6, HL-60, U937), MUTZ-3 most closely mimics DCs [27,28,29,31,34]. To differentiate into DC-like cells, MUTZ-3 appears to require TNF-α (our own observations and those of others [14,30,32,33]). Overall, there is a lot of discrepancy on the differentiation conditions of this cell line, especially on its dependency on TNF-α for differentiation. We and others [5] found the culture conditions of this particular cell line a challenging issue. Differentiated MUTZ-3 cells (expressing CD11c) did not show an immature DC status in any of our experiments or conditions. They rather displayed already a mature phenotype that could not be further stimulated. Contrary to Mo-DCs, differentiated MUTZ-3 cells could not effectively respond to the stimuli and we did not find differences between PBS-treated cells (our negative control) and cells stimulated with LPS or R848 in terms of surface marker expression. While this is in accordance with previous studies [9,33] that support the observed unresponsiveness of MUTZ-3, others have shown responses from this particular cell line upon stimulation with different components of *Haemophilus influenzae* [30]. Non-reactivity of MUTZ-3 cells to LPS stimulation has been linked to an impairment of the intracellular domain of the receptor (TLR4) or its interaction with the adaptor protein (MyD88) [33]. Additionally, MUTZ-3 cells have been shown to be selectively defective in their responses to pathogen-associated molecular patterns (PAMPs), but not to other types of stimulation (i.e., cytokines) which use different receptors and other intracellular signaling pathways [9]. These functional defects have been associated with the down-regulation of different gene clusters like those involved in pathogen recognition, DC maturation and cytokine/chemokine signaling in response to microbial stimulation [9]. We believe that dysregulation of these genes involved in TLR signaling pathways may explain why MUTZ-3 failed to respond to different ligands (LPS or R848) and to vaccines in our hands. The use of different readouts and stimuli different from ours (e.g., functional assays for antigen presentation and T cell stimulation) can explain why this cell line has proven useful to assess to a certain extent the biological and functional aspects of DCs [5,27,28,29,31]. Notwithstanding, for our particular purpose, MUTZ-3 proved not to be a suitable model for studying responses towards different vaccine formulations. 

The restricted ability of MUTZ-3 to respond to different stimuli prompted us to assess in a systematic way as to what degree DCs derived from PBMCs would be suitable as an in vitro vaccine screening platform. So far, human DCs have been mainly used to study vector [35,36,37,38], adjuvanted [30,35,39] or live attenuated vaccines [40,41] thus mainly focusing on the mechanism of action of the vector/adjuvant rather than on the vaccine itself. In contrast, we used next to TLR ligands two commercially available, inactivated and non-adjuvanted human vaccines known to differ in the responses they elicit in vivo [24,25]. Firstly, we demonstrate that in contrast to MUTZ-3 cells, Mo-DCs do respond to these vaccines; Secondly, we show that Mo-DCs respond to the vaccines in specific ways. The WIV vaccine activated Mo-DCs more effectively than the SU formulation. WIV efficiently elicited DC maturation, increasing the expression levels of surface and activation markers. The magnitude of the response was similar to and in some cases even higher than the magnitude of the response obtained by synthetic molecules like the TLR7 ligand, R848. Moreover, by using three different read-outs we found that WIV was capable of inducing upregulation of surface markers, and increased expression of genes relevant for the immune response and production of cytokines. The observed up-regulation of genes related to TLR signaling (*MYD88, IRF7*) further supports the notion that WIV stimulates Mo-DC by TLR7-triggering. The superiority of WIV over SU in activation of DCs has been previously shown by our lab for cultured DCs from mice [22]. Due to its composition, the WIV vaccine is intrinsically adjuvanted with viral ssRNA; this enables triggering of pattern recognition receptors (PRRs) like TLR7 and the subsequent activation of DCs [13]. Conversely, SU-treated cells did not display a surface marker phenotype consistent with mature/activated DCs, nor did they show any response in terms of gene expression. The SU vaccine is composed of proteins (HA and NA) that cannot be recognized by PRRs. This is consistent with the poor stimulation and low expression levels of surface markers observed by us.

Generally, the use of PBMCs or PBMC-derived cells is associated with the problem of donor variability and the fact that responses may be inconsistent across different readouts [11,42,43]. Recently, Li and colleagues [11] addressed the donor variability of immune responses to bacterial and fungal pathogens by assessing cytokine production. Regulation of the pathogen-specific responses was only partially genetically influenced, suggesting that external factors are also involved in the modulation of cytokine responses. In line with this, stimulation of MoDCs with WIV or SU did not show consistent effects on cytokine expression. From our results, we can conclude that not all readouts are suitable to pre-screen vaccine immunogenicity in this particular in vitro DC system. By combining the expression of surface/activation markers and specific genes as our main readouts we found consistent responses to the different vaccines across readouts and amongst the different donors. Additional extensive gene expression analysis could help to elucidate the mechanism of action of different vaccines. As for the production of cytokines, consistent vaccine-specific changes in expression were induced in the Mo-DCs, despite the inherent donor-to donor variation [11]. Nevertheless, the presence of low and high responders, which differ largely in the amount of cytokines they secrete, renders cytokine profiling a less suitable readout for the Mo-DC system. 

Another disadvantage of using primary Mo-DCs for vaccine assessment is the limited and time-restricted availability of PBMCs. However, when comparing Mo-DCs from fresh and frozen PBMCs, we observed that the capability of the cells to respond to different stimuli was not affected by the freeze/thaw process. This observation underlines that frozen PBMCs can be used for the generation of DCs, which largely enhances flexibility and reproducibility. Altogether, both the here reported consistency amongst donors and the possibility of using cryopreserved PBMCs highlight the feasibility of a primary DC-based in vitro system for the evaluation of vaccine candidates. 

Our data demonstrates that Mo-DCs are capable of responding to vaccines in measurable and consistent ways even if the vaccines are inactivated and non-adjuvanted. However, not all vaccines are capable of activating DCs. A good example of an adjuvant that does not induce DC responses is the oil-in-water compound AS03 that was approved for use in an integral part of pandemic influenza vaccines [44]. In view of this restriction there is a clear need to include additional types of immune cells to develop a cell-based in vitro system that allows a more extended assessment of vaccine immunogenicity and a detailed study of a vaccine’s mode of action.

In summary, during the differentiation to a DC-like phenotype, the MUTZ-3 cell line was already too activated and no further up-regulation of markers could be induced by any of the used stimuli. This characteristic renders the MUTZ-3 cell line an unsuitable model to mimic DCs. On the other hand, primary Mo-DCs, irrespective whether they were derived from fresh or frozen PBMCs, showed low variation amongst donors and proved to be a robust and reproducible platform to assess vaccine immunogenicity. We conclude that human Mo-DCs derived from frozen PBMCs are a flexible and robust platform for the evaluation of the immunogenicity of vaccine candidates and for the assessment of vaccine-induced immune mechanisms. 

## Appendix A

**Table A1:** Differentiation conditions for the MUTZ-3 cell line.

Days of Differentiation	IL-4 (U/mL)	GM-CSF (U/mL)	TNF-α (U/mL)
5	250	500	0
			250
		1000	0
			250
6	250	500	0
			250
		1000	0
			250
7	250	500	0
			250
		1000	0
			250
